# Application of the educational method of realistic simulation in the treatment of pressure injuries*

**DOI:** 10.1590/1518-8345.3946.3357

**Published:** 2020-09-07

**Authors:** Valéria da Silva Baracho, Maria Emília de Abreu Chaves, Thabata Coaglio Lucas

**Affiliations:** 1 Universidade Federal dos Vales do Jequitinhonha e Mucuri, Faculdade de Ciências Biológicas e da Saúde, Diamantina, MG, Brazil.; 2 Universidade Federal de Minas Gerais, Laboratório de Bioengenharia, Belo Horizonte, MG, Brazil.

**Keywords:** Wounds and Injuries, High Fidelity Simulation Training, Practice Patterns, Nurses’, Knowledge, Prevention & Control, Teaching, Ferimentos e Lesões, Treinamento com Simulação de Alta Fidelidade, Padrões de Prática em Enfermagem, Conhecimento, Prevenção & Controle, Ensino, Heridas y Traumatismos, Enseñanza Mediante Simulación de Alta Fidelidad, Pautas de la Práctica en Enfermería, Conocimiento, Prevención & Control, Enseñanza

## Abstract

**Objective:**

to evaluate the use of realistic simulation as a strategy to promote teaching about pressure injuries.

**Method:**

This is a quasi-experimental study. A modified and translated version of the *Pieper Pressure Ulcer* knowledge test was applied. Kappa statistical analysis was used to assess the professionals’ knowledge in the realistic simulation using the SPSS software. A *p*-value <0.05 was considered significant.

**Results:**

Seventy-seven nursing professionals participated in the realistic simulation, the majority (72.7%) being nursing technicians. Regarding the knowledge of primary and secondary coverage techniques, the Kappa index went from 0.56 (p=0.002) in the pre-test to 0.87 (p=0.001) in the post-test. As for the sterile dressing technique, there was a variation from 0.55 (p=0.002) in the pre-test to 0.91 (p=0.001) in the post-test. Regarding the cleaning of pressure injuries, there was a variation from 0.81 (CI: 0.62-0.84) in the pre-test to 0.91 (0.85-0.97) in the post-test. The knowledge about the use of a sterile spatula to distribute the dressing in the wound increased from an agreement index from *regular* to *good*.

**Conclusion:**

The introduction of the realistic simulation in the clinical practice has created quality assessment indicators for the prevention and treatment of pressure injuries.

## Abstract (pt)

**Objetivo:**

avaliar a utilização da simulação realística como estratégia para promoção do ensino sobre lesões por pressão.

**Método:**

trata-se de um estudo quase-experimental. Uma versão modificada e traduzida do teste de conhecimento *Pieper Pressure Ulcer* foi aplicada. Utilizou-se análise estatística de Kappa para avaliação do conhecimento dos profissionais na simulação realística por meio do software SPSS. Um valor *p*<0,05 foi considerado significativo.

**Resultados:**

setenta e sete profissionais de enfermagem participaram da simulação realística, sendo a sua maioria (72,7%) composta por técnicos de enfermagem. Em relação ao conhecimento das técnicas de cobertura primária e secundária, o índice de Kappa passou de 0,56 (p=0,002) no pré-teste para 0,87 (p=0,001) no pós-teste. Quanto à técnica estéril para curativo, houve uma variação de 0,55 (p=0,002) no pré-teste para 0,91 (p=0,001) no pós-teste. Quanto à limpeza das lesões por pressão, verificou-se uma variação de 0,81 (IC: 0,62-0,84) no pré-teste para 0,91 (0,85-0,97) no pós-teste. O conhecimento do uso de espátula estéril para distribuição da cobertura na ferida aumentou de um índice concordância *regular* para *bom*.

**Conclusão:**

a introdução da simulação realística na prática clínica criou indicadores de avaliação da qualidade quanto à prevenção e tratamento das lesões por pressão.

## Abstract (es)

**Objetivo:**

evaluar el uso de simulación realista como estrategia para promover la enseñanza sobre lesiones por presión.

**Método:**

estudio cuasi experimental. Se aplicó una versión modificada y traducida de la prueba de conocimiento *Pieper Pressure Ulcer*. El análisis estadístico de Kappa se utilizó para evaluar el conocimiento de los profesionales en simulación realista utilizando el *software* SPSS. Un valor de *p*<0,05 se consideró significativo.

**Resultados:**

setenta y siete profesionales de enfermería participaron en la simulación realista, con mayoría (72,7%) compuesta por técnicos de enfermería. Con respecto al conocimiento de las técnicas de cobertura primaria y secundaria, el índice Kappa pasó de 0,56 (p=0,002) en la prueba previa a 0,87 (p=0,001) en la prueba posterior. En cuanto a la técnica de apósito estéril, hubo una variación de 0,55 (p=0,002) en la prueba previa a 0,91 (p=0,001) en la prueba posterior. En cuanto a la limpieza de las lesiones por presión, hubo una variación de 0,81 (IC: 0,62-0,84) en la prueba previa 0,91 (0,85-0,97) en la prueba posterior. El conocimiento del uso de una espátula estéril para distribuir la cobertura de la herida aumentó de un índice de acuerdo de *regular* a *bueno*.

**Conclusión:**

la introducción de la simulación realista en la práctica clínica ha creado indicadores de evaluación de calidad para la prevención y el tratamiento de las lesiones por presión.

## Introduction

Pressure Injuries (PIs) represent a worldwide public health problem and their reduction is one of the goals of the World Health Organization. In the international scenario, the prevalence of PIs range from 5% to 39% and their incidence from 1.9% to 7.0%^(^ 1 ^-^ 4 ^)^. In Brazil, the incidence of PIs in Intensive Care Units (ICU) varies between 10.0% and 62.5%^(^ 1 ^-^ 5 ^)^.^^

In view of the epidemiological context of PIs, preventive measures to limit the spread of infections associated with injuries, the increase in hospital stay, financial costs, morbidity and mortality in health institutions generate quality indicators and reinforce the need to avoid adverse events ensuring patient safety^(^ 6 ^-^ 9 ^)^. The nurse has scientific and technological knowledge for an accurate assessment of wounds, being able to critically point out the risks and adverse events resulting from the failure to maintain the patient’s skin integrity^(^ 6 ^,^ 10 ^-^ 11 ^)^.

National and international guidelines indicate that simple preventive measures must be reinforced daily, such as: change of position at scheduled times, use of materials and therapies to provide pressure relief, predictive scales, adequate nutritional support, use of dressings, maintenance of the patient’s and bed’s hygiene, and health education not only for professionals, but also for family members^(^ 5 ^-^ 6 ^,^ 12 ^-^ 14 ^)^. Although the guidelines reinforce the use of preventive measures, in the clinical practice most of the health professionals do not adequately and rigorously provide care for the prevention of PIs.

Faced with this challenge, continuing education through innovative strategies of the pedagogical process, such as the use of active methodologies based on realistic clinical simulation, allows reproducing reliable situations of places and setting using scenographic objects, which contextualize the professional experience.

The main concern of the clinical simulation scenarios is to ensure that the professional reproduces aspects of reality in an interactive and dynamic way, and also reflects on the solutions of care problems based on scientific evidence, so that the simulation promotes a positive impact for the clinical practice^(^ 15 ^-^ 17 ^)^.

Health professionals move from being a listener to being a protagonist and autonomous of their own knowledge and begin to problematize various situations of care and to point out limitations and viable solutions for improving the quality and safety of health care^(^ 18 ^-^ 20 ^)^. 

Clinical simulations have their striking point in view of the discussions that take place after each scenario, in order to enhance learning through experience, called debriefing. This is considered a moment of self-reflection and discussion about the performance of the team member in the face of the activity developed. Thus, it is possible to highlight critical points of the scenario, positive aspects of the colleague’s performance in order to clarify what to do differently when evaluating the performance made by the “volunteer actor” of the realistic clinical simulation^(^ 16 ^-^ 17 ^,^ 19 ^)^.

In view of the above, the following is asked: “How can realistic clinical simulation influence the improvement of the teaching-learning process of nursing professionals?” Considering this gap in scientific knowledge about a new pedagogical teaching strategy for nursing professionals and students based on problematization regarding the care and treatment of PIs, the objective was to evaluate the use of the realistic simulation as a strategy to promote teaching about PIs.

## Method

A quasi-experimental study, which was carried out in a philanthropic health institution in the inland of Minas Gerais/Brazil from April to December 2018. The study population consisted of nursing professionals, namely: nurses, nursing technicians and assistants, in addition to undergraduate nursing students working in the hospitalization clinics of the study institution. 

The inclusion criteria were the following: being a professional working in one of the hospitalization clinics (medical, surgical, neurological, and health insurance). Nursing professionals who worked in the emergency room, intensive care unit, and hemodialysis were excluded from the study. In addition, professionals who were on vacation or on leave were excluded from the study.

The recruitment and selection of research participants took place through the monthly service schedule, made available by the technician responsible for Nursing. The professionals were divided into teams, two during the day and two at night, so as not to compromise the work routine and patient care. 

A random probabilistic sample was used, and the sample size was calculated using the Epidat software, version 4.1 (Pan American Public Health Organization), setting the following parameters: expected proportion of 50%, due to the heterogeneity of the variables to be measured; tolerable error margin of 5% and 95% confidence level. The sample size was adjusted for finite populations and 30% was added to the defined number to compensate for possible losses or refusals, and 20% to increase the statistical power of the proportion comparison tests.

The groups of participants were invited to participate in the study regardless of gender, age, working time or training. The total population of the participating clinics consisted of 18 nurses, 69 nursing technicians, and 10 nursing students. The sample, however, was composed of 77 workers, considering the exclusion criteria. In addition, five nursing technicians and three nurses refused to participate in the research.

To implement data collection, four phases were followed:

Phase I: pre-intervention period (a): prior to the simulation, the researcher presented the implementation of the new active methodology in the health institution, not only for the theme related to the PIs, but also for all the themes associated with the patient’s clinic. The principles and objectives of the realistic clinical simulation were explained so that the professionals were comfortable when the activity started.

To evaluate the knowledge of the professionals about wounds, a semi-structured questionnaire was applied, pre-test, from the *Pieper/Pressure Ulcer Knowledge Test*(PUKT)^(^ 8 ^,^ 23 ^-^ 24 ^)^. The adapted and validated version for Brazil was used^(^ 25 ^)^. This phase of applying the pre-test lasted two months.

The original instrument contained 47 items that measured knowledge of prevention, risk, stage, and description of the wound. The test included true and false questions. Correct answers received 1 point and incorrect answers received no points. International studies verified the effectiveness of PUKT in educational programs for Nursing and showed to be effective^(^ 8 ^,^ 24 ^)^. Although the PUKT questionnaire evaluates the specific knowledge of the nurse, for this study, the variables were adapted to a simple and easy language so that the content involved the basic theoretical knowledge, important for the care of the PIs both for students and the entire nursing team. The adapted questionnaire aimed to develop critical and reflective aspects with the entire team involved with the prevention and care of PIs in the daily practice, regardless of specific knowledge in each category; therefore, we emphasize that it did not involve educational actions for Nursing technicians to learn private actions of nurses, such as the prescription of dressings. In the intervention of this study, the purpose of four dressings specifically used in the institution stood out, such as hydrogel, hydrogel with alginate, calcium alginate and collagenase, which often generated doubts among the entire team, regarding the appropriate cleaning techniques of the wound bed, and specific care of the dressing after its closure.

The questionnaire applied had 13 true and false statements. The variables presented to the entire nursing team were the following: assessment of decubitus change, function of the dressing, ideal healing environment, evaluation of the PI, cleaning, dressings, stage of the PI, aseptic dressing technique and use of personal protective equipment.

The pre-test was prepared by the authors themselves and applied to the research participants on the day of the simulation, with a duration of 15 minutes for everyone. The researcher requested the non-identification of the participants and that the questions should not be discussed among them until the end of the simulation. 

In addition, four images of PIs containing the following tissues were presented in the questionnaire: “epithelium”, “granulation”, “slough”, and “necrotic tissue”. The participants wrote in full what types of tissues were presented in the images.

In order to assess the knowledge of the teaching methodology, there was the following question in the pre-test: “*Do you know the teaching methodology of realistic simulation?”* It was categorized descriptively as “yes” or “no”.

Phase II: pre-intervention period (b): clinical cases were applied to the professionals and the problematization of the PIs related to prevention, care, and treatment was discussed. The second phase lasted five hours with each team of participants for the day and happened during a period of three months. It was held in a private room, provided by the study site. The participants were asked not to share the experience of the clinical cases with other groups that had not yet participated in this phase of the study. 

Each team was composed of five professionals, chosen at random, and there was a total of three meetings, that is, three days of discussion, so that they could reflect on the clinical cases and resume them in the following days with new arguments. In addition, all the professionals who participated in Phase I also participated in Phase II.

Phase III: intervention phase (c): The realistic simulation was performed with a mannequin and reusable artificial wounds. A simulation scenario was also created with two Nursing students previously trained by the researchers to simulate clinical cases associated with PIs. The selected volunteer professionals performed the task of the selected clinical cases according to the knowledge discussed in Phase II. This Phase lasted two months so that all professionals could be trained. The scenario started with a summary of approximately 10 minutes, followed by an explanation of the simulation’s objectives that involved care and treatment with PIs and the introduction of clinical cases. The first professional started the initial simulation session of 20 minutes. After the first session, a 7-minute interrogation session was conducted by two members of this research, both are registered nurses with a clinic experience of more than 5 years, including PI care. The simulation sessions were alternated so that each of the five participants in the group, chosen at random, could be an actor in the simulation. Overall, the simulation scenario lasted 80 minutes, so that there was time to exchange experiences and discuss the cases presented.

The following materials were used for the scenario: kidney dishes, sterile dressing kit, gauze, gloves, 0.9% saline solution, bandage, adhesive tape, dressings, and artificial blood.

The description of the simulations was based on the learning objectives, with a focus on the development of clinical reasoning for the evaluation and treatment of PIs. In order to achieve the objectives outlined in each scenario, the heterogeneity of each clinical case was determined, with relevant information for the participants to interpret and make the necessary associations in the face of different themes, such as: identification of wound stages, tissue characteristics, implementation of the treatment from cleaning the injury to the application of adequate topical therapy, as well as interventions that prevent new injuries and that favor the tissue repair process.

Phase IV: post-intervention (d): each clinical case scenario was conducted by one of the researchers in this study who, after the closure, performed the debriefing. This was structured by two questions addressed to the other participants: *1) What positive actions were taken? 2) What would you do differently on another occasion?*

At this moment, a “diagnosis” of the action performed was promoted together with the participants, that is, everything that was done was analyzed and discussed. The researcher assumed the position of facilitator of the group discussion, by working on the feelings experienced by the participants who performed the simulations, identifying the successes and opportunities for improvement, as well as promoting diagnostic/therapeutic reasoning, critical thinking, and judgment. 

Phase V: After completing the realistic simulation experiment, the same questionnaire from Phase I was applied. In addition, in the post-test, there was one more question regarding the self-assessment of the professional performance in relation to dressing before using the realistic simulation as a learning method. It was categorized descriptively as “*bad”, “regular”, “good”*, and *“excellent”*. 

The collected data were categorized and analyzed using the *Statistical Package for the Social Sciences* ^®^(SPSS) software, version 20. The unweighted Kappa agreement index with a 95% confidence interval was used. The significance level was set at p<0.05. Kappa values range from -1 (total lack of agreement) to 1 (total agreement). The conventional interpretation of Kappa value is: 0.00-0.20 = poor agreement; 0.21-0.40 = regular; 0.41-0.60 = moderate; 0.61-0.80 = good; and 0.81-1.00 = very good.

The reliability of the responses was analyzed and compared with the answers of three experts on the subject (gold standard), compared with the results presented by the nursing team and students. Both the pre-test and the post-test were submitted to the analysis of the judges, in which the three specialists were invited to judge the items of the questionnaire as to relevance, adequate and clear language, and whether the theory presented met both the Nursing technicians and the Nursing students involved in care at the institution. The questionnaire for analysis by the judges was sent via e-mail along with the invitation letter and the Free and Informed Consent Form (FICF). Items that did not obtain a level of Kappa agreement and Content Validity Index (CVI) among the judges of K≤0.81 and CVI≤0.80 were removed.

The research participants were invited to collaborate with the study and, after clarifying the objective, those who agreed signed the FICF. This research was approved by the Research Ethics Committee of UFVJM, under protocol No. 3,144,715.

## Results

Seventy-seven Nursing professionals participated in this study, the majority (72.7%) being Nursing technicians (Table 1). The working time of the Nursing professionals ranged from 1 to 5 years (70.13%), from 5 to 10 years (20.78%), and over 10 years (9.09%).

**Table 1:** Distribution of the category of the nursing professionals and students who were included in the study. Diamantina, MG, Brazil, 2018

Professional category	n (%)
Nurse	10 (13)
Nursing technician	56 (72.7)
Nursing assistant	5 (6.5)
Student	6 (7.8)
Total	77 (100)

With regard to the participants’ prior knowledge in relation to the realistic simulation teaching methodology, 68 (88.31%) answered that they did not know this teaching strategy and 9 (11.69%) that they already knew it. Table 2 shows the Kappa agreement index in the assessment of the knowledge of the research participants regarding prevention, treatment, and infection control measures associated with the technique.

**Table 2:** Distribution of the knowledge test statements according to the Kappa agreement index related to prevention, treatment and infection control measures in the control of pressure injuries. Diamantina, MG, Brazil, 2018

Statements from the knowledge test	Pre-test	Kappa Index	Post-test	Kappa Index
	95% CI*	(Standard Deviation)	95% CI*	(Standard Deviation)
	(p-value)^†^		(p-value)^†^	
A scale with times for changing decubitus is required only for patients with PI^‡^, not for those at risk. (F)^§^	-0.23.-1.00 (p=0.002)	0.90 (0.021)	-0.32.-1.00 (p=0.001)	0.94 (0.032)
The dressing has the following functions: to keep the wound moist, to remove exudate, and to allow gas exchange. (T)^||^	0.43.-1.00 (p=0.002)	0.87 (0.032)	-0.21.-1.00 (p=0.001)	0.98 (0.0012)
The ideal environment for the healing of PIs^‡^ is occlusive and dry. (F)^§^	0.73.-0.93 (p=0.002)	0.80 (0.018)	0.65.-1.00 (p=0.001)	0.95 (0.009)
The assessment of PI^‡^ for signs of infection is not always important. (F)^§^	0.95.-0.99	0.97 (0.0014)	0.95.-1.00 (p=0.001)	0.99 (0.001)
For cleaning the PIs^‡^ 0.9% SS^¶^ is applied in a jet heated to 35-37ºC. (T)^||^	0.62-0.84 (p=0.002)	0.81 (0.032)	0.85-0.97 (p=0.001)	0.91 (0.002)
The alginate hydrogel is applied to very exudative, sloughing, bleeding, and open wounds. (F)^§^	0.34.-1.00 (p=0.002)	0.61 (0.023)	0.62.-0.98 (p=0.001)	0.94 (0.012)
Alginate and collagenase are applied to granulation tissue. (F)^§^	0.55.-0.81 (p=0.002)	0.75 (0.032)	0.88.-1.00 (p=0.001)	0.93 (0.031)
After the primary dressing, the use of a secondary one is recommended, as it avoids the risk of moisture or contamination. (T)^||^	0.55.-0.81 (p=0.002)	0.56 (0.021)	0.81.-0.98 (p=0.001)	0.87 (0.034)
PIs^‡^ in Stage II can be painful due to the exposure of nerve endings. (T)^||^	0.41.-0.57 (p=0.002)	0.43 (0.023)	0.62.-0.85 (p=0.001)	0.76 (0.017)
Granulation tissue is formed by the proliferation of vascular endothelial cells and fibroblasts. (T)^||^	0.47.-0.64 (p=0.002)	0.56 (0.021)	0.89.-0.97 (p=0.001)	0.95 (0.011)
It is recommended to use a non-sterile spatula to distribute the dressing on the wound bed. (F)^§^	0.23.-0.45 (p=0.002)	0.30 (0.045)	0.57.-0.89 (p=0.001)	0.76 (0.035)
Personal protective equipment such as apron and mask are not recommended during dressing. (F)^§^	0.51.-0.68 (p=0.002)	0.62 (0.031)	0.76.-0.97 (p=0.001)	0.93 (0.032)

* CI = 95% Confidence interval;

† p-value <0.05;

‡ PI = Pressure injury;

§ F = False;

|| T = True;

¶ SS = 0.9% Saline solution

As for the written identification by the research participants, of four types of images of different tissues present in the PIs, Table 3 shows the distribution of the answers according to the Kappa index.

**Table 3:** Distribution of the variables to identify the types of tissues present in pressure injuries, according to the Kappa index. Diamantina, MG, Brazil, 2018

Variables	Pre-test 95% CI* (p-value)^†^	Kappa Index (Standard Deviation)	Post-test 95% CI* (p-value)^†^	Kappa Index (Standard Deviation)
Epithelial tissue	0.46.-0.61	0.56	0.56.-0.87	0.78
	(p<0.001)		(p=0.001)	
Granulation tissue	0.47.-0.71	0.55	0.75.-0.91	0.86
	(p<0.01)		(p<0.001)	
Slough	0.11.-0.45	0.21	0.75.-0.91	0.91
	(p=0.002)		(p<0.001)	
Necrosis	0.71.-0.92	0.87	0.95.-0.99	0.97
	(p=0.001)		(p=0.001)	

* CI = 95% Confidence interval;

† p-value<0.05

Regarding the professionals’ self-assessment of performance in the dressing technique before they knew the realistic simulation teaching method, 48 (62.3%) considered their performance to be *regular*, 17 (22.1%) *good,* 8 (10.4%) *excellent*, 1 (1.3%) *bad*, and 3 (3.9%) did not answer.

## Discussion

This study proposed a new teaching methodology that was reproducible and effective not only for the study institution, but also for other health care establishments as a model for teaching and training.

In view of the results obtained, it was verified that the level of knowledge of the nursing professionals and students, associated with the Kappa concordance index, was mostly *very good*, after performing the realistic simulation with values greater than 90. Such agreement value represents the acquisition of an adequate level of knowledge for the institution^(^ 4 ^,^ 8 ^,^ 24 ^)^. 

Regarding the other statement in the questionnaire, “the use of a non-sterile spatula for the distribution of the dressing on the wound bed”, there was a significant improvement in agreement (p<0.05), going from *regular*(0.30) to *good*(0.76). Despite not reaching an ideal level of agreement, there was an improvement in care with the maintenance of the proper environment in the preparation of the dressing in order to prevent the infection of a wound.

A cross-sectional study carried out in Australia used the Pieper test to assess nursing knowledge in relation to PIs^(^ 24 ^)^. Although the study did not evaluate an improvement in knowledge after a certain intervention, it was verified that most of the responses associated with the prevention and treatment of PIs were considered satisfactory (79%), not reaching the ideal knowledge that would be greater than 90% according to the scale model used in the study^(^ 24 ^)^. 

In general, the realistic clinical simulation contributed to the increase of the Kappa index and, consequently, to the increase of the knowledge and attitudes of the nursing team in the face of the challenges of daily practice involving the care with the PIs.

This change in attitude is justified, since it was evaluated what the professionals knew (Phase II) and how they performed prevention, care, and treatment in the practice (Phase III). This schedule was essential for changing the behavior of the institution, since the professionals evaluated themselves, mainly because it is a new method for most nursing professionals (88.31%).

The realistic simulation method was the subject of a study that demonstrated the importance of this method in nursing teaching and learning through a narrative literature review^(^ 21 ^)^. The mentioned study verified that simulation has great educational importance as it enhances joint learning, interaction among teams, communication, and the exchange of knowledge among professionals as they identify in practice the positive points of the colleague and what they would do differently in care.

Through an experience report, a number of studies analyzed the construction and development of realistic simulation scenarios for the training of Nursing professionals, including the stage and the scenario for debriefing^(^ 18 ^-^ 19 ^)^. The authors verified that the use of the simulation technique was a new experience in the health institution, allowing the participants to approach and reflect on the “way of doing” from a new educational approach, in which knowledge and experiences were shared. In addition, the need was highlighted to invest in the construction of scenarios based on real facts and the use of in-service simulation proposals, involving topics related to patient safety and investing in the qualification of health care professionals.

In the present study, debriefing was a key point for the development of the capacity to rescue critical-reflective reasoning about PIs, as it contributed to the creation of a positive self-image of the professional and to a better improvement of skills and abilities during care practices.

A meta-analysis study carried out in Iran analyzed several European and Australian surveys that used PUKT to assess Nursing knowledge in relation to PIs^(^ 22 ^)^. It was verified that the total percentage of knowledge in relation to the prevention of PIs was 53.1% (95% CI: 47.5-58.8), which would be acceptable but not desirable^(^ 22 ^)^. These data corroborate the results of the pre-test of this study, which showed that sufficient knowledge about the prevention and care of PIs is not influenced by geographic regions. In the countries evaluated, it was verified that the Nursing team did not have the knowledge based on scientific evidence desirable for an ideal care plan in the prevention of PIs.

Regarding the identification of images of the different tissues present in the PIs, despite the significant improvement (p<0.05) of knowledge after the practice of the realistic simulation, it was verified that the professionals still had some difficulty in knowing what type of tissue they were taking care of in their daily practice. Knowledge of the types of tissues such as epithelium, granulation, slough, and necrosis was considered essential in the knowledge of the Nursing team because it is related to the application of bandages and dressings which, if not correctly evaluated, can impair the treatment of PIs.

A cross-sectional study, carried out in Istanbul, assessed the knowledge of the Nursing team through a questionnaire adapted from PUKT^(^ 8 ^)^. The authors observed that, of the Nursing professionals eligible for the study, 180 (58.4%) had a score over 60% and 128 (41.6%) obtained values below 60% of the correct answers^(^ 8 ^)^. The study concluded that the Nursing knowledge was insufficient and that there would be a risk in the quality of the care plan for the prevention of PIs. As in Brazil, the high incidence of PIs made the government support effective actions for its prevention and treatment^(^ 8 ^,^ 26 ^)^.

Assessing the knowledge and attitudes of the professionals in their practice contributes to determining educational priorities and to the development of specific interventions within health institutions^(^ 24 ^,^ 27 ^)^.

A cross-sectional study carried out in Belgium assessed the knowledge of nurses and Nursing technicians about the care with PIs through the PUKT test^(^ 11 ^)^. The mean number of correct answers regarding issues of care and prevention of PIs was 50.7%^(^ 11 ^)^. The theoretical knowledge of the Nursing team was considered inadequate^(^ 11 ^)^.

In addition, participants who were present in training on PIs had a higher percentage of correct answers when compared to those who did not participate in the training (61.0% *versus* 50.2%, p=0.004)^(^ 11 ^)^. These results corroborate with the data of the present study, since higher values of agreement of the Kappa index were observed in the post-test of the professionals after participating in the realistic simulation. Through an active teaching dynamics, even pathophysiological concepts can be established, as verified in this study through the identification of different types of tissues (Table 3). These concepts are essential for determining an effective preventive measure and for preventing recurrent treatments of PIs.

One of the limitations of this research was that, although the study covered a period of nine months, the post-test questionnaire was applied immediately after the realistic simulation, which may have influenced the improvement in the knowledge of the professionals. However, the period between Phases II and IV lasted three months, so that the professionals could study and become familiar with the clinical cases and with the problematization of the themes. Future exploratory studies could be carried out with the entire multidisciplinary team to compare the knowledge of PIs among the different professional categories.

## Conclusion

The educational strategy of realistic simulation used in this study proved to be effective for improving the knowledge of PIs and changing the behavior of the professionals in the Nursing practice, especially as it is a marker of quality of care in which nurses play a fundamental role. The results of this study came from a modified version of PUKT and indicated significant gaps in knowledge about the prevention and treatment of PIs.
